# Secondary Students’ Knowledge on Birds and Attitudes towards Conservation: Evaluation of an Environmental Education Program

**DOI:** 10.3390/ijerph20105769

**Published:** 2023-05-09

**Authors:** Unai Ortega-Lasuen, Oier Pedrera, Erin Telletxea, Oihana Barrutia, José Ramón Díez

**Affiliations:** 1 Department of Didactics of Mathematics, Experimental and Social Sciences, University of the Basque Country (UPV/EHU), 48940 Leioa, Spain; tellerata@gmail.com (E.T.);; 2 Department of Didactics of Mathematics, Experimental and Social Sciences, University of the Basque Country (UPV/EHU), 20018 Donostia San Sebastian, Spain; oier.pedrera@ehu.eus (O.P.); oihana.barrutia@ehu.eus (O.B.)

**Keywords:** biodiversity, outdoor education, bird migration, species identification, environmental attitudes

## Abstract

Urdaibai Biosphere Reserve holds a diversity of habitats and resources that are essential for migratory bird species’ conservation, and at the same time provides a rich milieu for the development of environmental education programs. This study evaluates the impact of a daylong and place-based environmental education program, performed at the Urdaibai Bird Center (UBC), on secondary education students’ environmental attitudes and knowledge. Students (*n* = 908) completed a written questionnaire where their perceptions on the Urdaibai Biosphere Reserve and marshes, together with their interest in biodiversity, knowledge about bird migration and bird species identification skills, and attitudes towards conservation were assessed. Results show that students’ knowledge regarding Biosphere Reserves, marshes, and bird migration is limited, and that their bird identification skills are scarce. Although they scored high on environmental attitudes, a significant number of them feel that conservation efforts are excessive and hinder economic development. Students living within the Biosphere Reserve, as well as from rural milieus or who underwent primary education through a bird-centered curriculum hold a better knowledge of the local biodiversity. To adapt the environmental education program at the UBC, its integration in formal teaching/learning contexts via meaningful hands-on and/or project-based activities could be considered, together with the systematic evaluation of the outcomes.

## 1. Introduction

According to the Intergovernmental Science-Policy Platform on Biodiversity and Ecosystem Services [1], nature is declining at an alarming rate worldwide. The functioning of ecosystems, and consequently the services provided by them, are underpinned by biodiversity to a large extent [2,3]. Hence, this human-driven loss of nature and biodiversity is of major concern since it poses a direct threat to human wellbeing globally [4].

In this paradigm, environmental conservation stands out as the way to go in order to reduce biodiversity loss and achieve the Sustainable Development Goals (SDGs) [5]. However, environmental issues go beyond development and ecological problems, since they are bound by how nature and environment are understood, particularly in Western civilization. Indeed, according to previous studies, environmental knowledge seems to partly influence both environmental attitudes and behaviors [6,7]. Hence, considering that laypeople have limited environmental literacy [8], an essential requirement to address this illiteracy and consequently improve the environmental situation lies largely in the education and literacy of the citizenship, with Environmental Education (EE) being an indispensable instrument to reshape the way people think of the environment [9]. However, EE is not a mere tool for environmental problem-solving [10], but an essential dimension of basic education focusing on all three domains of learning (i.e., cognitive, affective, and behavioral) and key to empowering people to make informed and responsible decisions regarding their environmental behavior [11].

In this attempt to improve people’s environmental literacy, increase awareness, and foster action, multiple EE approaches have been employed in both formal and informal contexts [12]. Some of the most recurrent teaching/learning strategies in EE over the years have been the ones that endeavor to bring nature closer to citizens, such as outdoors learning, field experiences, and out of school activities [13], viewing connectedness with nature as a precursor of environmental literacy [14]. In this sense, protected natural areas are often considered sites that offer unique and excellent opportunities for developing outdoors and place-based EE programs [15,16]. In particular, Biosphere Reserves represent learning laboratories for sustainable development, as they integrate functions such as the conservation of biodiversity and cultural diversity, socio-culturally and environmentally sustainable economic development, and logistical support underpinning development through research, monitoring, education, and training [17].

Although EE has been suggested to be effective in the instruction and literacy of citizens on environmental topics [18], most EE programs lack systemic evaluation [19]. Thus, even though they are generally described as great opportunities to create an environmentally literate society, without proper evaluations, the effectiveness of these programs is contingent at best [20]. This hinders identifying and assessing the factors that affect and influence the acquisition of environmental knowledge and the development of attitudes and behaviors within EE programs [21,22], making it difficult to modify these programs so that they can meet their objectives.

In addition, most EE assessments are conducted right after implementing EE programs, or over a short period of time [19,23,24], making the information regarding the retention of the outcomes, or the long-term effects of these programs, scarce. Therefore, although environmental educators may have a biased and not an evidence-based measure of the impacts of their EE programs [22,25], the positive outcomes they perceive seem to fade away within a short period of time, particularly when the interventions are of short duration [26,27].

Regarding environmental literacy, species identification skills are emphasized as essential prerequisites for comprehending biodiversity and ecology [28], and their achievement is closely related to sustainable and pro-environmental attitudes [29,30]. Härtel et al. [31] provide evidence on the influence of species knowledge on both environmental system knowledge and attitude towards the environment in secondary students, as high species knowledge proved to have a positive effect on their participants’ attitudes towards the environment. However, identification skills among laypeople are deficient, especially regarding native species [32,33,34]. According to Hooykaas et al. [35] this lack of awareness is particularly concerning in the case of birds, as people fail to identify even some of the most common species in their homeland. In a survey conducted with Bavarian adults, Enzensberger et al. [36] described lower bird identification scores among young participants. Additionally, this illiteracy is also reflected in several misconceptions regarding the anatomy of birds, their behavior (e.g., migration), and bird–human interactions [37,38,39].

Birds are ubiquitous animals of great ecological importance as several bird species contribute to ecosystem functioning and provide ecosystem services such as pest control, seed dispersal, pollination, scavenging carcasses, and nutrient cycling [40]. However, bird communities are following the same declining tendency as other biodiversity globally [41,42]. The main reason for this population and biodiversity decline is the loss of suitable habitats and their connectedness due to anthropic pressures [43]. Further, this is of particular concern when it comes to wetlands [44], since these threatened, fragile, and vulnerable ecosystems host great species richness, and several globally threatened birds depend on them as they serve as important habitats for birds to breed, nest, and rear young among other functions [45].

Therefore, considering that conservation efforts are futile without fostering a meaningful scientific and ecological literacy in the citizenship, the objectives of this study are dual. On the one hand, this research intends to diagnose and characterize the knowledge and attitudes secondary students have regarding birds and marsh conservation depending on different sociocultural factors, and on the other hand, it intends to analyze the effects that undergoing a punctual place-based EE program which takes place in the Urdaibai Biosphere Reserve (UBR), a protected natural space dominated by one of the best-preserved estuaries on the Cantabrian coast, can have on these aspects, and how much these effects last. Thus, this research addresses the following questions:

1. What perceptions do secondary students have regarding Biosphere Reserves and marshes? What is their interest in biodiversity?
2. What knowledge do these students have on bird migration and migrant bird species at the UBR?
3. What attitudes towards the UBR’s conservation do these students hold?

## 2. Materials and Methods

### 2.1. Study Site

The research took place at nine different secondary schools that had previously participated in the EE program at the Urdaibai Bird Center (UBC). These schools are located near the Urdaibai estuary in Bizkaia (Basque Autonomous Community, Northern Spain, 43°22′ N 2°41′ W), a Cantabrian catchment of 23,000 ha declared a Biosphere Reserve by UNESCO in 1984.

This region of temperate oceanic climate is of great natural interest as it is home to more than 3000 species of flora and fauna, including some highly endangered species [46]. Furthermore, this RAMSAR and Birds Special Protection Area is especially important for biodiversity conservation, as it contains a diverse mosaic of habitats and serves as a stop for migratory bird species [47]. The UBC observatory is located next to the Orueta lagoon, where daily observations of the birds present are recorded on the eBird platform (https://ebird.org/hotspot/L2779555/media?yr=all&m=, accessed on 26 April 2023). These observations have resulted in the reporting of more than 235 bird species on this citizen science platform, contributing information on their frequency and distribution throughout the year.

However, in spite of being a protected area and presenting better conservation features than neighboring regions [48], conflicts between conservation and economic interests have emerged in the region [49].

### 2.2. Participants and EE Program

908 students from compulsory secondary education (SE1 to SE4), ranging from 12 to 15 years of age, participated in this study. Eight of the nine participant schools were located within the UBR (Busturialdea region), while one school was from an adjacent district. All the educational centers took part at some point in the Osprey Project for EE at the Urdaibai Bird Center (UBC), a museum and birdwatching point located in the marshes of the UBR dedicated to the study and scientific divulgation of birds and their migration (https://www.birdcenter.org/en, accessed on 26 April 2023).

The EE program in which some students participated lasts about 2 and ¼ h, and the main questions that configure the program are:

- What is a marsh? Why is it so important?
- How do I identify a bird?
- How can I see birds in their natural habitat?
- How and why do we monitor birds?
- How can I help protect birds and marshes?
- Why is migration so dangerous?
- Why do I need green spaces?
- What skills have I acquired? What new knowledge do I have?

The program starts with a guided visit to the museum in which the visitors get explanations about the phenomenon of migration and the importance of the UBR. Afterwards, videos and images about the Osprey Project in which the UBC takes part are shown. Hereafter, the habitats of the UBR are shown and explained in the observatory at the museum, underlining the characteristics and importance of marshes. At this point students have the opportunity to watch and identify different bird species using binoculars. The visit continues with an outdoor walk to another observatory. Along the way different explanations regarding birds and habitats of the UBR are given, and the visit ends with a bird identification game at the museum observatory.

### 2.3. Research Instrument

**Figure 1:**
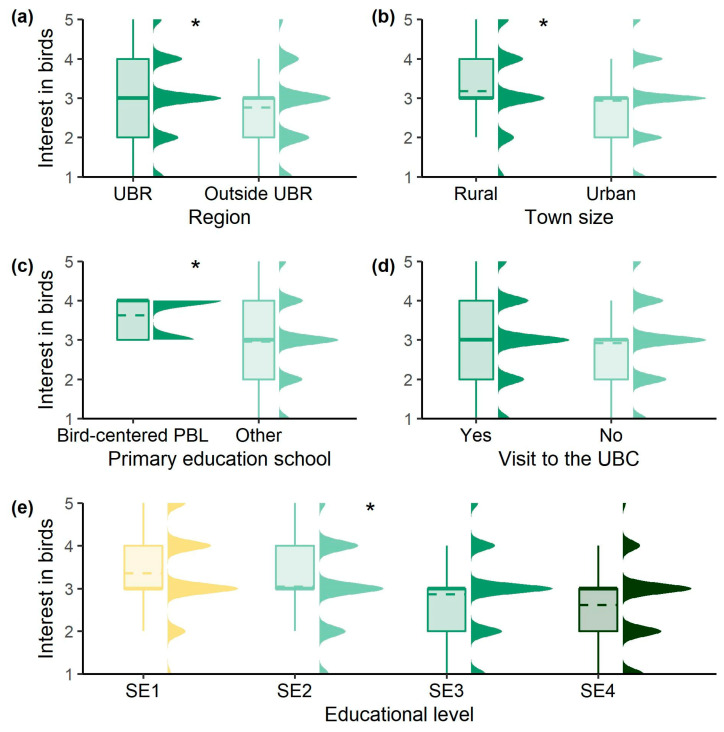
Students’ interest in birds (*Q14*) regarding the factors studied: (**a**) RR, (**b**) TS, (**c**) PES, (**d**) PV and (**e**) EL. Asterisk stands for significant differences (*p* < 0.05) and dashed lines represent the mean.

The time that had elapsed between the visit and the completion of the questionnaire was variable, ranging from 1 to 4 years, as the school groups could have visited UBC during both primary and secondary school. Students completed a written questionnaire in their respective classrooms for 40 min, under the supervision of their teachers. This questionnaire consisted of six parts including single-choice, multiple-choice, Likert-scale, and open questions (Table 1): (1) Characterization of the students (Q1–Q7); (2) Perceptions on the UBR and marshes (Q8–12); (3) Interest in biodiversity (Q13–Q14); (4) Knowledge about bird migration (Q15–Q16); (5) UBR bird species identification skills and knowledge evaluation with a task (Figure 1) where students had to identify and name ten common bird species (i.e., mallard (*Anas platyrhynchos*), Eurasian coot (*Fulica atra*), common kingfisher (*Alcedo atthis*), great cormorant (*Phalacrocorax carbo*), little egret (*Egretta garzetta*), barn swallow (*Hirundo rustica*), Eurasian spoonbill (*Platalea leucorodia*), osprey (*Pandion haliaetus*), greylag goose (*Anser anser*) and Eurasian curlew (*Numenius arquata*)) and determine whether they could be observed in UBR and whether they were migrant species (Q17); and (6) Attitudes towards the conservation of the UBR environment (Q18–Q22). The questionnaire’s English version is presented in the Supplementary Materials.

For the bird identification task (Q17), species were selected and agreed upon with the environmental researchers from UBC on the basis of their encounter rate and representativeness in the EE program at UBC (in photographs, diagrams, explanations, etc.), as they are commonly observed and identifiable during the outdoor walk. These species are present in the reserve throughout the year and are easily accessible on sight. The barn swallow and osprey are present from March to October–November, and the greylag goose from September to May. The osprey is the only species with an intermediate ease of sighting, as it is observable in specific areas (Table 2).

All the necessary ethical considerations were respected in the design and implementation of the questionnaire, and written consent forms from the parents or legal guardians of the students, school directors, and teachers were collected after they had been duly informed about the objectives and procedures of the research.

### 2.4. Data Analysis

The results obtained with the written questionnaire were analyzed using IBM SPSS Statistics v. 24. Different variables were defined as independent based on the answers of the students (Table 3).

Thus, two categories were defined for the region of residence: residents within the region where the UBR is located (Busturialdea) and residents outside the UBR region. Likewise, town size was considered by establishing 10,000 inhabitants as the threshold to differentiate rural and urban towns, as defined by the local statistical agency (EUSTAT) to differentiate between rural and urban municipalities for the Basque Country. Gernika and Bermeo, the main towns within the UBR region, have 16,808 and 16,910 inhabitants, respectively, while the surrounding municipalities range from having 235 to 1843 inhabitants [52].

Similarly, two levels were defined for primary education school, differentiating those students who performed primary education at two local schools that conduct their curriculum by means of bird-centered Project Based Learning (PBL). These two schools also work alongside the UBC in a project named “The birds around us” with the objective of bringing nature and birds close to children and instilling scientific competencies.

Moreover, in the question regarding bird species identification (Q17), each answer was given a numeric value depending on the grade of correctness. Thus, an identification score (ID score) was calculated [53], where correct identifications of the species (e.g., mallard) received 1 point, halfway correct identifications (e.g., duck) received 0.5, and incorrect answers got 0.

In addition, among the students that had previously visited UBC, the exact dates of their previous visits were known in 182 cases. Thus, the days that had passed from the visit to the fulfilment of the questionnaire were determined, and these were found to be ranging from 377 to 1251 days (1 to 3.5 years). Linear regressions were conducted to determine the retention capacity of species identification skills.

For Q18 to Q22, the mean score, representing attitudes towards the conservation of the UBR environment, was calculated for the correlation analysis.

The Mann–Whitney U test and Kruskal–Wallis rank test were performed for ordinal data. On the other hand, the Chi-square test (χ2) was applied for categorical data. Correlations were calculated using Spearman’s correlations.

## 3. Results

### 3.1. Perceptions on the UBR and Marshes, and Interest in Biodiversity

Students’ responses regarding their self-perceived knowledge of UBR and marshes significantly varied within most of the studied variables (Table 4). Students living in the UBR region claimed a significantly higher understanding of what a Biosphere Reserve was. On the other hand, residents from smaller towns, who had completed their primary education through bird-centered PBL, had previously visited UBC, and had enrolled on higher educational levels (SE3–SE4 vs. SE1–SE2) reported better understanding of Biosphere Reserves and marshes.

Furthermore, when asked about the importance of the UBR, most of the students (58%) agreed that it hosted a high natural and cultural richness in a relatively small area. Similarly, 69% underlined the determinant role that the UBR plays in bird migration, and 11% indicated that the reason for its importance lay in its touristic attraction. Moreover, regarding the importance of marshes, 49% of the students correctly identified their productivity as the reason for their importance and 63% acknowledged their importance for water birds. Besides, 43% believed that conservation and economic development should be equally promoted. However, although the majority accepted that the UBR was of great importance and must be conserved, 16% thought that the conservation measures were too strict, and 24% opined that conservation was prioritized over the interests of the inhabitants.

Regarding the students’ interest in plants and animals, 52% reported being interested in both types of living organisms, 35% showed interest in animals exclusively, 2% claimed being interested in plants solely, and 10% answered being interested in neither animals nor plants. Moreover, on average, students showed a medium interest in birds, as 46% felt indifference towards them. However, 26% of the respondents showed high or very high interest in birds, and it is worth noting that the students who were interested in both animals and plants showed higher interest in birds than the rest of the students (Z_U_(890) = 7.843, *p* < 0.001).

Furthermore, this interest in birds significantly varied among most of the different variables studied, following similar tendencies to the ones observed regarding self-perceived knowledge (Figure 1). Thus, although in this case having previously visited the UBC did not lead to significant differences, students living within the UBR (Z_U_(872) = −2.264, *p* = 0.024), from smaller towns (Z_U_(872) = 2.403, *p* = 0.016), who had completed bird-centered PBL in primary education (Z_U_(884) = −3.165, *p* = 0.002), and of lower educational levels (Z_KW_(3) = 63.860, *p* < 0.001) reported higher interest in birds.

### 3.2. Knowledge about Bird Migration and Species Identification

The questions about bird migration revealed some misconceptions among secondary students, mainly with respect to the reasons for this phenomenon. Almost half of the students (48%) answered that birds uniquely migrate looking for hotter temperatures, disregarding the role of migration in the search for new resources. Students that previously underwent the EE program at the UBC identified looking for resources as the main reason better than those who had not visited the UBC (Z_χ2_(1) = 104.632, *p* < 0.001).

Comparably, students from lower educational levels answered more frequently that the main reason for migration was temperature (Z_χ2_(3) = 14.473, *p* = 0.002), while the ones from higher educational levels chose more often the correct answer related to resources (Z_χ2_(3) = 16.735, *p* = 0.001). On the other hand, most of the students (91%) correctly identified that each bird species has a different migratory destination.

**Figure 2:**
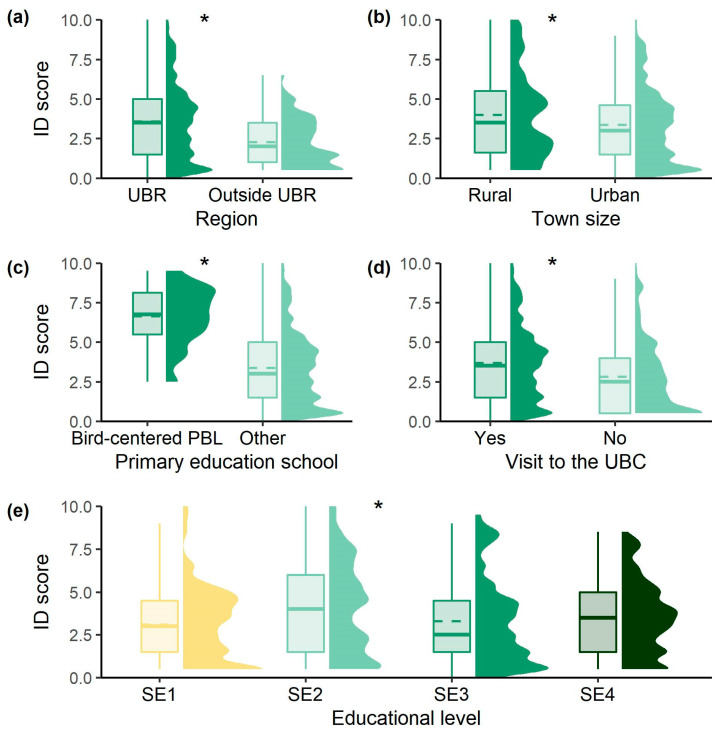
Students’ bird species identification scores (*Q17*) regarding the factors studied: (**a**) RR, (**b**) TS, (**c**) PES, (**d**) PV and (**e**) EL. The asterisk stands for significant differences (*p* < 0.05) and dashed lines represent the mean.

Students’ identification skills were proven scarce as the average score for identification was 3.42 ± 0.09. However, statistical differences were present within all of the studied factors (Figure 2); students from the UBR region (Z_U_(790) = −4.621, *p* < 0.001), living in smaller towns (Z_U_(790) = 2.105, *p* = 0.035), who completed their primary education through bird-centered PBL (Z_U_(803) = −4.835, *p* < 0.001), and who had visited the UBC (Z_U_(809) = −4.622, *p* < 0.001) scored higher in the bird identification task. Similarly, SE2 stood out from the rest of the educational levels (Z_KW_(804) = 14.652, *p* = 0.002).

**Figure 3:**
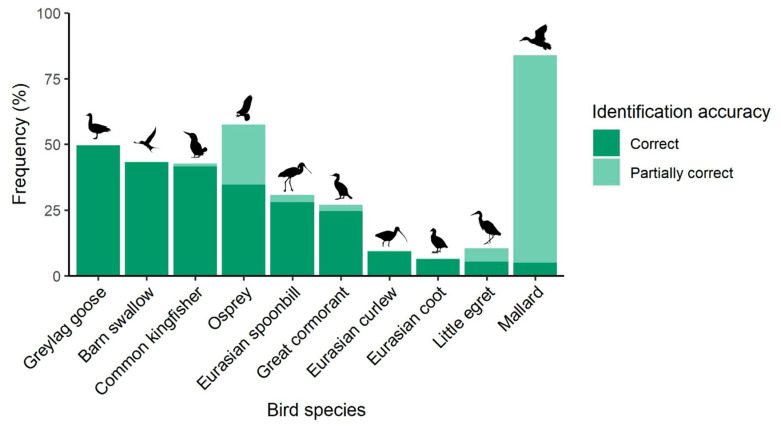
Students’ correct and halfway-correct identification percentages for ten bird species present in the UBR.

Furthermore, regarding correctly identified species, the most correctly recognized were the greylag goose (50%), barn swallow (43%) and common kingfisher (42%) (Figure 3). However, only 5% of students correctly named a species as widespread and common as the mallard, even if 79% identified it halfway with putative names such as ‘duck’, ‘blue duck’, or ‘green head duck’. This phenomenon was also visible in the case of the osprey, as 35% identified it correctly and 23% partially correctly by naming it simply ‘raptor’.

**Figure 4:**
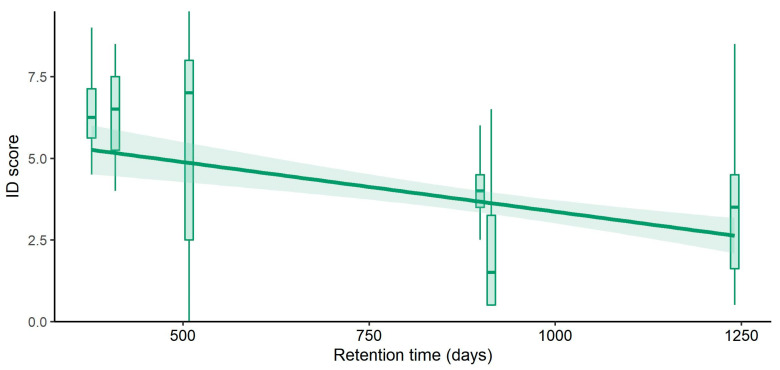
Boxplot and regression line for bird identification score and the time passed from conducting the EE program at UBC and fulfilling the bird identification task in the questionnaire (Retention Time, *n* = 182).

Besides, a moderate negative correlation was recorded between the time that had passed since undergoing the EE program at the UBC and the bird identification score (*r_s_*(182) = −0.333, *p* < 0.001), revealing that bird species identification skills had significantly decreased with time (Figure 4).

In addition, all the queried species can be found in the UBR at least during some periods of the year, but only five out of the ten are migratory: the barn swallow, Eurasian spoonbill, osprey, greylag goose, and Eurasian curlew. This way, the mean of correct answers regarding their presence in the UBR was 5.98 ± 0.10, and the migratory nature of these species was only correctly identified on average in 2.64 ± 0.06 of the cases. Students from the UBR region (Z_U_(790) = −2.298, *p* = 0.022), who had previously visited the UBC (Z_U_(808) = −4.871, *p* < 0.001), of SE2 (Z_KW_(805) = 17.381, *p* = 0.001), and especially those from the selected primary schools (Z_U_(804) = −3.966, *p* < 0.001) showed a higher ability to correctly assess whether these species could be found in the UBR or not. On the contrary, the migratory nature of these species was better acknowledged by those who had studied primary education through the bird-centered PBL curriculum (Z_U_(671) = −1.994, *p* = 0.046), and those from SE2 and SE3 educational levels (Z_KW_(673) = 22.182, *p* < 0.001).

### 3.3. Attitudes towards the UBR’s Conservation

**Figure 5:**
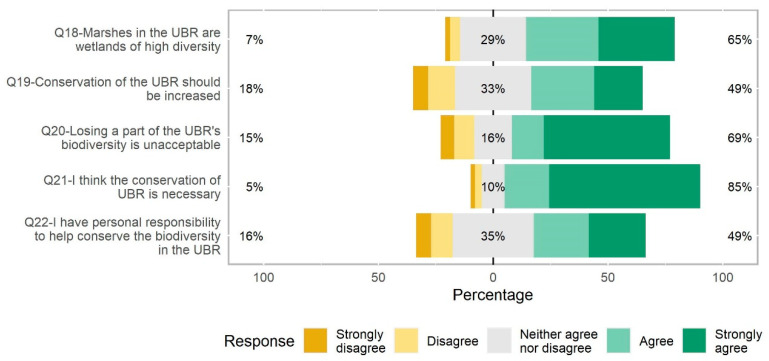
Students’ responses to Likert-type questions regarding conservational attitudes towards the UBR (Q18–Q22).

Students presented relatively high punctuations regarding environmental attitudes (Figure 5). Overall, 60% agreed to an extent that the UBR’ marshes were high biodiversity areas, 63% did not find it acceptable to lose a part of their biodiversity, and 79% believed that the UBR’s conservation was a must. However, it is also worth noting that 15% of them thought that they had no personal responsibility in the conservation of the UBR’s biodiversity, and 17% considered that the UBR’s conservation level and measures should not be increased.

Besides, regarding environmental attitude, those who had previously visited the UBC (Z_U_(853) = −4.347, *p* < 0.001) and those from lower educational levels (Z_KW_(848) = 21.088, *p* < 0.001) presented higher environmental values.

Finally, it is worth underlining that environmental attitudes positively correlated with the number of correctly identified bird species (*r_s_*(855) = 0.295, *p* < 0.001), number of correct answers regarding whether these species could be found in the UBR (*r_s_*(771) = 0.176, *p* < 0.001), self-perceived knowledge on marshes (*r_s_*(853) = 0.240, *p* < 0.001), self-perceived knowledge on Biosphere Reserves (*r_s_*(850) = 0.326, *p* < 0.001), number of correct answers in the questions about bird migration (*r_s_*(855) = 0.219, *p* < 0.001) and interest in birds (*r_s_*(847) = 0.249, *p* < 0.001).

## 4. Discussion

The present study provides evidence on how different factors such as the place of residence and town size, the integration of EE in the curriculum through PBL, and undergoing punctual EE programs affect and shape secondary students’ perceptions on Biosphere Reserves and marshes, knowledge on migration and bird identification, and environmental attitudes.

Natural protected areas in general, and Biosphere Reserves in particular, besides being high biocultural diversity areas useful for conservation and the development of the SDGs, also provide great educational opportunities for place-based learning [16,17]. Most students agreed that both the UBR and its marshes are rich areas of high natural interest, comprehending their importance for bird migration and ecosystem productivity. However, although almost half of the students showed sustainable attitudes by answering that both conservation and economic development should be promoted in the UBR, several students manifested dissatisfaction with the conservation measures as they found them too restrictive and against the common interest of the inhabitants. These opinions could be influenced by their social context, not only because environmental values are partly influenced by families [54], but also because similar results were documented in a local survey showing a general lack of knowledge regarding what Biosphere Reserves are and the importance of their conservation [55].

Besides, even though students revealed being interested in biodiversity, plants were dismissed in almost half of the cases: a well-documented behavior [33,56]. Moreover, although students reported interest in animals and most of them lived in a natural enclave for migratory birds, the average interest in avifauna was intermediate, in accordance with previous works [57,58].

Regarding bird migration, the same misconception described by Prokop et al. [39] and Cardak [37] arose, as many believed that the purpose of migration was the search for suitable temperatures instead of the foraging for resources scarce on bird breeding grounds. However, the vast majority of the students answered correctly that each species had a specific migration path and destination.

Moreover, despite birds being practically ubiquitous and charismatic animals, students’ identification of common birds in the UBR area was poor. It is remarkable that, despite the fact that students may use generic or unspecific names to refer to different animal taxa (e.g., duck, eagle, etc.), the greylag goose and barn swallow were, together with the common kingfisher, the most recognized species. This may be related to different local cultural variables in the Basque Country, such as the fact that in the local language these are not compound names, the popularity of the goose festival is considerable, and *Enara* (a common name for the barn swallow in the Basque language) is a popular proper noun.

Additionally, the students also failed at recognizing which species could be found in the UBR and explaining their migratory nature, showing overall limited knowledge regarding native bird species. These results are in line with prior research stating that species knowledge is scarce among young people [34,59,60].

Otherwise, students’ environmental attitudes towards the UBR were relatively high. However, even if they were a minority, some respondents showed less pro-environmental opinions, and this was particularly concerning, bearing in mind their lack of awareness of the consequences of one’s actions and their poor conceptualization of biodiversity’s importance, both of which negatively impact biodiversity conservation due to little willingness to act and protect it [61].

Hopefully, students showing higher knowledge about bird species identification, migration, and marshes also revealed more pro-environmental attitudes. Although this relationship is widely documented in the literature [62,63,64,65], inferring a causal relationship between these two variables is hard due to the complexity of the factors affecting both cognitive and affective domains, and even more difficult considering the environmental behavior and willingness to act [66]. However, it is fair to assume that these two are at least linked to some extent and should be promoted considering their influence on environmental behavior [6,60]. 

Regarding the sociocultural variables, the results among the local residents of the UBR and from smaller and more rural towns suggest that direct contact with nature has a direct positive effect on local biodiversity knowledge [67,68,69,70]. This is in accordance with the so-called “extinction of experience” phenomenon, of particular concern regarding children, and characterized by an ever-increasing alienation from the natural environment due to the rapid urbanization and spread of sedentary and virtual leisure activities [71]. Furthermore, some works have described that bird species identification skills are related to nature connectedness [72] and that attitudes towards birds vary along the urbanization gradient [73]. Students from more rural regions tend to show more biophilic attitudes and are more prone and willing to support animal conservation than urban children [74,75]. Indeed, Otto & Pensini [7] affirm that connectedness to nature is a better predictor of environmental attitudes and behavior than any other factor, and Randler and Heil [76] identify birding specialization and bird-related interest and activities as the most important predictors of bird species literacy.

The importance and advantages of outdoor learning and EE have been widely documented in the literature as they are effective in the instruction and literacy of citizens, generally having positive outcomes in at least one of the three domains: cognitive, affective, and behavioral [77,78,79]. The results of the current study are in accordance with the abovementioned statement and demonstrate that EE programs, regardless of their duration, are useful in enhancing environmental knowledge and attitudes at least in the short/mid-length term [24]. However, it is worth noting that some studies defend the idea that punctual EE programs may have ephemeral effects on students [80,81], a phenomenon that has also been observed in this study, highlighting that the retention time of this kind of program is limited and far from being lifelong [10]. 

Moreover, it is noteworthy that the observed improvement of interest towards birds and, in particular, knowledge, was even more noticeable in the case of the students that had completed their primary education in local schools that conduct their curricula through bird-centered PBL and work alongside UBC. Therefore, this finding suggests that although a punctual EE program can help in enhancing knowledge, attitudes, and behavior at least within a short time span, a long-term and well-planned intervention comprising active learning methodologies is more likely to be associated with both a better retention and a higher increase in ecological literacy [21,26].

Finally, students from higher educational levels presented better self-perceived knowledge of Biosphere Reserves, marshes, and bird migration, and lower-educational-level students had greater interest in birds and higher environmental attitudes. However, the students from the SE2 level were the ones who best identified and knew local bird species. These observations align to some extent with previous works suggesting that species identification skills are powerful during childhood but tend to decrease during puberty [82] since the identification of organisms and their names no longer seem important to youngsters [83]. Additionally, the higher environmental interest and attitudes of younger students from this study can be explained due to a higher connection with nature and curiosity [84], making them more receptive and responsive to EE than adolescents [85,86]. Hence, special educational efforts have to be conducted during compulsory secondary education in order to keep motivated and literate young students in biodiversity.

The main limitations of the present study are related to the low sample size for RR, PES, and TS factors, which reduces the sensitivity of the statistical analysis, specially for PES. Further research considering wider samples is needed to evaluate the implications of these factors on the outcomes of daylong EE programs aimed at secondary school students. Moreover, the approach to long-term evaluation, especially in terms of bird species identification skills, should be considered with caution as other longitudinal aspects have not been considered.

## 5. Conclusions

The findings in this study lead to several implications. First, they illustrate that secondary students’ factual knowledge regarding bird migration is limited, and particularly that their bird identification skills are scarce. Second, they show that although teenagers’ environmental attitudes are high, a significant number of them consider that conservation efforts are excessive and an obstacle to economic development, both of which are seemingly inherited conceptions. Further, all these variables are affected by different sociocultural factors, with students living in the Biosphere Reserve and with rural backgrounds holding a better knowledge of the local biodiversity, and students that underwent primary education through an EE-centered curriculum showing a better grasp of them. 

It can be concluded that although EE programs can be a great and useful way to enhance the appreciation of protected natural areas and foster meaningful environmental knowledge and attitudes at least in the short term, the daylong EE program performed at the UBC presents a limited effectiveness. To adapt this program, its curricular integration in formal teaching/learning contexts via meaningful hands-on and/or project-based activities could be considered, together with the systematic evaluation of its outcomes.

## Figures and Tables

**Table 1:** Items of the questionnaire, type of question and references.

Number	Description	Type	Reference
(1) General information (Q1–Q7)			
Q1	Gender	Single choice	-
Q2	Secondary education school	Open	-
Q3	Educational level	Single choice	-
Q4	Region of residence	Open	-
Q5	Hometown	Open	-
Q6	Primary education school	Open	-
Q7	School visit to the UBC	Single choice	-
(2) Perceptions on the UBR and marshes (Q8–Q12)			
Q8	Self-perceived knowledge on Biosphere Reserves	Likert scale	-
Q9	Opinion on conservation measures at the UBR	Multiple choice	-
Q10	Importance of the UBR	Multiple choice	-
Q11	Self-perceived knowledge on marshes	Likert scale	-
Q12	Importance of UBR’s marshes	Multiple choice	-
(3) Interest in biodiversity (Q13–Q14)			
Q13	Interest in UBR’s plants and animals	Single choice	[29] *
Q14	Interest in birds	Likert scale	
(4) Knowledge about bird migration (Q15–Q16)			
Q15	Reasons for bird migration	Multiple choice	[37,39]
Q16	Bird migration destination	Multiple choice	
(5) UBR bird species identification skills and knowledge (Q17)			
Q17	Common UBR bird species identification, presence and migrant nature	Open	[34,50] *
(6) Attitudes towards conservation of the UBR environment (Q18–Q22)			
Q18	Biodiversity richness	Likert scale	[51] *
Q19	Increase of conservation measures	Likert scale	
Q20	Acceptance of biodiversity loss	Likert scale	
Q21	Need for conservation	Likert scale	
Q22	Personal commitment to conservation	Likert scale	

* means modified from ref.

**Table 2:** Main characteristics of the species presented in Q17: migrant pattern (R: resident/sedentary; S: migrant summer resident; W: migrant winter resident), observation seasons in UBR (Green: most favorable; Orange: possible; White: not present) and difficulty of observation (E: easy, accessible on sight; I: intermediate, in specific areas).

Species		Migrant Pattern	Observation Seasons	Observation Difficulty
Mallard	*Anas platyrhynchos*	R	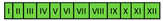	E
Eurasian coot	*Fulica atra*	R	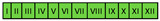	E
Common kingfisher	*Alcedo athis*	R	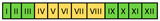	E
Great cormorant	*Phalacrocorax carbo*	R	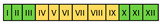	E
Little egret	*Egretta garcetta*	R	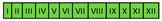	E
Barn swallow	*Hirundo rustica*	S	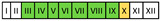	E
Eurasian spoonbill	*Platalea leucorodia*	S	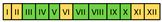	E
Osprey	*Padion haliaetus*	S	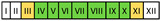	I
Greylag goose	*Anser anser*	W	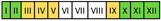	E
Eurasian curlew	*Numerius arquata*	W	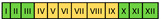	E

**Table 3:** Characteristics of the student sample according to different variables.

Variable	Category	Sample Size (*n*)
Gender (G)	Female	467
	Male	404
Region of Residence (RR)	UBR	791
	Other	92
Town Size (TS)	Rural	121
	Urban	762
Primary Education School (PES)	Bird-centered PBL	16
	Other	880
UBC Previous Visit (PV)	Yes	653
	No	254
Educational Level (EL)	SE1 (Age 12)	235
	SE2 (Age 13)	185
	SE3 (Age 14)	280
	SE4 (Age 15)	200

**Table 4:** Students’ answers to the questions about their self-perceived knowledge of Biosphere Reserves (Q9) and marshes (Q12) including the mean and standard error of the mean ($\bar{x}$ ± SEM), Z-scores for the Mann–Whitney U test ^1^, and *p*-values.

		Q9-What Is Your Knowledge on Biosphere Reserves?				Q12-What Is your Knowledge on Marshes?			
Var.	Cat.	$\bar{x}$ ± SEM	df	Z	*p*	$\bar{x}$ ± SEM	df	Z	*p*
RR	UBR	3.53 ± 0.04	874	−3.757	<0.001	3.39 ± 0.04	877	−0.679	0.331
	Other	3.10 ± 0.11				3.33 ± 0.11			
TS	Rural	3.74 ± 0.09	874	2.613	0.009	3.62 ± 0.10	877	2.288	0.022
	Urban	3.45 ± 0.04				3.35 ± 0.04			
PES	PBL	4.06 ± 0.20	888	−2.168	0.030	4.38 ± 0.20	889	−3.833	<0.001
	Other	3.48 ± 0.04				3.37 ± 0.04			
PV	Yes	3.59 ± 0.04	898	−4.770	<0.001	3.50 ± 0.04	900	−4.906	<0.001
	No	3.19 ± 0.07				3.05 ± 0.08			
EL ^1^	SE1	3.46 ± 0.08	3	40.159	<0.001	3.37 ± 0.08	3	38.164	<0.001
	SE2	3.08 ± 0.08				2.94 ± 0.09			
	SE3	3.60 ± 0.06				3.59 ± 0.06			
	SE4	3.72 ± 0.07				3.49 ± 0.07			

^1^ The Z-score regarding Educational Level stands for Kruskal–Wallis’ H statistic.

## Data Availability

The data presented in this study are available on request from the corresponding author.

## Supplementary Materials

The following supporting information can be downloaded at: https://www.mdpi.com/article/10.3390/ijerph20105769/s1, Student’s Questionnaire.
