# Supplementary material for: Secondary Students’ Knowledge on Birds and Attitudes towards Conservation: Evaluation of an Environmental Education Program
Source: Int J Environ Res Public Health. 2023 May 9;20(10):5769. doi: 10.3390/ijerph20105769 (PMC10218049; doi:10.3390/ijerph20105769)
Supplement: Supplementary file 1 [file ijerph-20-05769-s001.zip › ijerph-2343565-supplementary.pdf]

International Journal of *Environmental Research and Public Health*

MDPI

Supplementary Materials

Secondary Students' Knowledge on Birds and Attitudes towards Conservation: Evaluation of an Environmental Education Program

Unai Ortega-Lasuen [1,*], Oier Pedrera [2], Erin Telletxea [1], Oihana Barrutia [2] and José Ramón Díez [1]

[1] Department of Didactics of Mathematics, Experimental and Social Sciences, University of the Basque Country (UPV/EHU), 48940 Leioa, Spain
[2] Department of Didactics of Mathematics, Experimental and Social Sciences, University of the Basque Country (UPV/EHU), 20018 Donostia San Sebastian, Spain
* Correspondence: unai.ortega@ehu.eus; Tel.: +34-946-014-560

STUDENT´S QUESTIONNAIRE

- **Girl or boy?** ....
- **School:** ....
- **Course:** ....
- **Hometown:** ....
- **What was your school in Primary Education?** ....
- **Have you ever visited Urdaibai Bird Center?** ☐ Yes ☐ No
- **With whom you visited?** ☐ Family ☐ School ☐Others: …………..
- **If you visited it with School, have you repeated?** ☐ Yes ☐ No
- **What is a Biosphere Reserve?** (Pick all the options you think are right)
  ☐ I've never heard that word before
  ☐ I don't quite understand it, but I've heard the word sometime
  ☐ I understand a little
  ☐ I understand pretty well
  ☐ I get it right and I can explain it to a friend
- **In your opinion in the Urdaibai Biosphere Reserve…** (Pick all the options you think are right)
  ☐ conservation and development are equally encouraged
  ☐ conservation measures are very strict
  ☐ conservation takes precedence over the interests of the people
  ☐ the interests of the people are above conservation
  ☐ I don´t know

- **Why is Urdaibai Biosphere Reserve important?** (Pick all the options you think are right)
  ☐ Because of the diversity of habitats in a small area
  ☐ Because it plays a crucial role for migratory birds in making their way
  ☐ Because it attracts a lot of tourists
  ☐ I don't think Urdaibai Biosphere Reserve is important
  ☐ I don´t know

- **What is a marsh?** (Pick all the options you think are right)
  ☐ I've never heard that word before
  ☐ I don't quite understand it, but I've heard the word sometime
  ☐ I understand a little
  ☐ I understand pretty well
  ☐ I get it right and I can explain it to a friend

- **Why are they important Urdaibai´s marshes?** (Pick all the options you think are right)
  ☐ Because marshes are very productive ecosystems
  ☐ Because they are necessary for waterbirds
  ☐ Marshes are not important
  ☐ I don´t know

- **Are you interested in animals and/or plants that live in Urdaibai?** (Just pick one answer)
  ☐ Animals ☐ Plants ☐ Animals and plants ☐ Neither

- **What is your interest in birds?** (Just pick one answer)
  ☐ Very little ☐ Little ☐ Halfway ☐ Big ☐ Very big

- **Why do some birds migrate?** (Just pick one answer)
  ☐ They just migrate for heat
  ☐ They migrate for food
  ☐ Birds don't migrate

- **Where do birds migrate?** (Just pick one answer)
  ☐ All birds migrate to the same place
  ☐ Every bird species has its route, some go to Africa
  ☐ Birds don't migrate

- **Fill in the next table:**

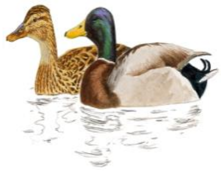	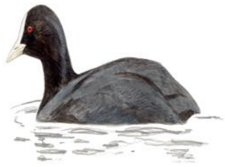	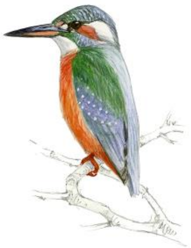	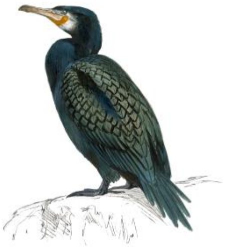	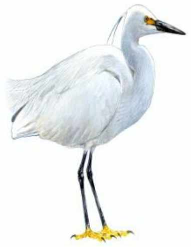
**Name: ……………… ……**	**Name: ……………… ……**	**Name: ……………… ……**	**Name: ……………… ……**	**Name: ……………… ……**
**Can it be seen in Urdaibai?** ☐ Yes ☐ No	**Can it be seen in Urdaibai?** ☐ Yes ☐ No	**Can it be seen in Urdaibai?** ☐ Yes ☐ No	**Can it be seen in Urdaibai?** ☐ Yes ☐ No	**Can it be seen in Urdaibai?** ☐ Yes ☐ No
**Does it migrate?** ☐ Yes ☐ No	**Does it migrate?** ☐ Yes ☐ No	**Does it migrate?** ☐ Yes ☐ No	**Does it migrate?** ☐ Yes ☐ No	**Does it migrate?** ☐ Yes ☐ No
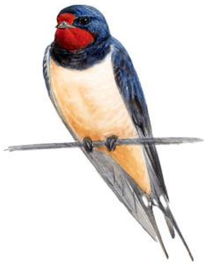	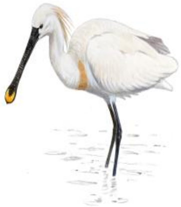	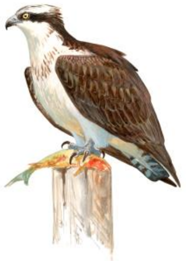	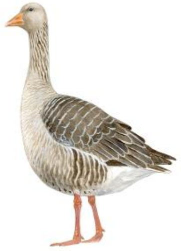	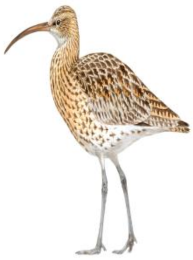
**Name: ……………… ……**	**Name: …………… ………**	**Name: ……………… ……**	**Name: ……………… ……**	**Name: ……………… ……**
**Can it be seen in Urdaibai?** ☐ Yes ☐ No	**Can it be seen in Urdaibai?** ☐ Yes ☐ No	**Can it be seen in Urdaibai?** ☐ Yes ☐ No	**Can it be seen in Urdaibai?** ☐ Yes ☐ No	**Can it be seen in Urdaibai?** ☐ Yes ☐ No
**Does it migrate?** ☐ Yes ☐ No	**Does it migrate?** ☐ Yes ☐ No	**Does it migrate?** ☐ Yes ☐ No	**Does it migrate?** ☐ Yes ☐ No	**Does it migrate?** ☐ Yes ☐ No

- **Value the following sentences from 1 (TOTAL DISAGREEMENT) to 5 (TOTAL AGREEMENT) and indicate with an X the option you think best:**

Marshes in the Urdaibai Biosphere Reserve are wetlands of high biodiversity	1	2	3	4	5
Conservation of the Urdaibai Biosphere Reserve should be increased	1	2	3	4	5
Losing a part of Urdaibai Biosphere Reserve´s biodiversity is acceptable	1	2	3	4	5
I think that the conservation of Urdaibai Biosphere Reserve is necessary	1	2	3	4	5
I have a personal responsibility to help in the conservation of Urdaibai Biosphere Reserve´s biodiversity and its endangered species	1	2	3	4	5

Thank you very much for your collaboration!
